# Missed Care Prevalence and Associated Factors in Acute Care Settings: A Systematic Review and Meta‐Analysis

**DOI:** 10.1111/ijn.70097

**Published:** 2026-01-09

**Authors:** Afia Achiaa Sarpong, Lucy Gent, Amanda Towell‐Barnard, Diana Arabiat

**Affiliations:** ^1^ School of Nursing and Midwifery Edith Cowan University Perth Western Australia Australia; ^2^ Centre for Nursing Research, Sir Charles Gairdner Hospital Perth Western Australia Australia; ^3^ Aflac Cancer and Blood Disorders Center, Children's Healthcare of Atlanta at Hughes Spalding Atlanta Georgia USA; ^4^ Maternal and Child Nursing Department, Faculty of Nursing The University of Jordan Amman Jordan; ^5^ Epidemiology, Biostatistics and Health Informatics Department, Public Health Institute (PHI) The University of Jordan Amman Jordan

**Keywords:** care left undone, meta‐analysis, missed, nursing care, patient outcomes, prevalence

## Abstract

**Aim:**

The aim of the study was to comprehensively analyse the prevalence and factors associated with missed nursing care in acute care settings.

**Methods:**

A systematic review was conducted to estimate the prevalence of missed nursing care, types of activities missed and associated factors in acute care hospital settings. Five electronic databases (CINAHL, Embase, Medline, PubMed and Scopus) were searched from inception to 14th December 2022. Type of missed care and associated factors were classified based on missed care concepts, definition and measurement. Random effects meta‐analysis was performed to estimate proportions and levels of types of nursing activities missed.

**Results:**

A total of 45 studies recruited 139 454 nurses reporting missed care activities. The most frequent activity missed was ambulation, with estimated prevalence of 46% (95%, CI [0.37, 0.55] *I* ^2^ = 99.6), followed by mouthcare 36% (95% CI [0.30, 0.43], *I* ^2^ = 99.7%), emotional support 33% (95% CI [0.26, 0.43], *I* ^2^ = 99.5%), bathing 31% (95% CI [0.22, 0.41], *I* ^2^ = 99.6%) and feeding 30% (95% CI [0.23, 0.38], *I* ^2^ = 99.5%). Limited workforce capacity was a major contributor to missed care.

**Conclusions:**

This study reports significant missed basic nursing activities in healthcare settings globally, suggesting greater odds of unsafe patient outcomes of hospitalisation. Targeted preventative strategies need to be tailored to specific missed care activities.

## Summary

**What Is Already Known About This Topic?:**

- Missed nursing care is an important health problem impacting health care systems across the globe.
- Inadequate nurse staffing and material resources are two important reasons for missed nursing care occurrence.

**What This Paper Adds?:**

- Meta‐analysis showed that the most frequently missed nursing activities reported across the globe were found to be within the domain of fundamental or basic, care.
- Nurses are frequently unable to fully provide for hospitalised patients' care needs such as ambulation, mouthcare, emotional support, bathing and feeding.

**The Implications of This Paper:**

- This study contributes to nursing knowledge and practice, indicating the need for awareness creation and/or educational programs on missed nursing care for both nurses and consumers.
- Strengthening nursing capacity, involving nurses in addressing missed care and establishing preventative systems are recommended to help reduce missed care and its associated risks.

## 1 Introduction

Missed nursing care (MNC) has been recognised by many international scientists as an extremely challenging healthcare phenomenon linked to unsafe patient outcomes (Jones et al. 2021). In 2015, the global prevalence of MNC ranged from 55% to 98% (Jones et al. 2015). With the progression of MNC, patients worldwide gradually face increased risk of falls, pressure injury and hospital‐acquired infections (Mandal et al. 2020). MNC can severely affect the quality of life of patients and increase disease management burden for the patient, their family and community (Palese et al. 2015). The phenomenon of MNC was first described by Aiken et al. (2001) as a ‘task left undone.’ Kalisch (2006) independently further developed the term ‘missed nursing care’ and described the phenomenon as any delay or omission of nursing care either partially or fully. Other terms used in the literature interchangeably with MNC include ‘implicitly rationed nursing care’ (Schubert et al. 2007), ‘care left undone’ (Ausserhofer et al. 2014) and ‘unfinished nursing care’ (Bayram et al. 2024; Jones et al. 2015; Sochalski 2004). A recently published book on the theory and research on MNC has highlighted the lack of clarity and uniformity in the concept and terminologies of MNC (Jones et al. 2021; Papastavrou and Suhonen 2021). This study used Kalisch’s term MNC to refer to the occurrence of this phenomenon.

Kalisch (2006) summarised the types and extent of MNC including ambulation, turning, delayed or omitted feedings, patient teaching and patient discharge planning. Research evidence from hospital settings has shown that nurses more frequently missed basic care elements (such as bathing, emotional support and communication) and less frequently missed technical care elements (such as medication administration and patient preparation for a procedure) (Blackman et al. 2018; Higgs et al. 2020; Jones et al. 2015). MNC has been linked to a reduced nurse workforce and can potentially lead to negative patient outcomes (Ball et al. 2018; Chaboyer et al. 2021; Cho et al. 2021). As a response to the rampant increase in the occurrence of MNC in healthcare systems in the past few decades (Sarpong et al. 2023), there has been enhanced focus on tackling this phenomenon from multiple perspectives (Papastavrou and Suhonen 2021). Despite growing interest in improving safe patient outcomes, MNC continues burdening patients and the healthcare systems (Ausserhofer et al. 2021).

Although previous systematic reviews have indicated a relatively high occurrence of MNC (Amrolahi‐Mishavan et al. 2022; Bassi et al. 2018; Jones et al. 2015; Kalánková et al. 2019; Nilasari and Hariyati 2021; Ogboenyiya et al. 2020; Rahmah et al. 2022), there is a paucity of systematic reviews of research investigating the prevalence of MNC (Mandal et al. 2020). As the systematic review and meta‐analysis is a scientific approach that helps obtain global integrated syntheses of different studies, findings from these studies can lead to reasonably targeted interventions (Ahn and Kang 2018). Despite the importance of such evidence in global nursing education and practice, at this present time, to the best of our knowledge, no systematic review and meta‐analysis has systematically assessed the evidence on the types and levels of reported MNC. Therefore, there is a need to determine the prevalence of MNC in healthcare systems. This study aimed to comprehensively analyse the prevalence and factors associated with MNC in acute care settings. Specific objectives were also to identify the types of MNC that are most reported by nurses and to examine factors that may be associated with the prevalence of MNC.

## 2 Methods

The protocol for this systematic review and meta‐analysis was registered online with PROSPERO and is available online (CRD42021256837). This review is reported in accordance with the PRISMA (Page et al. 2021). The JBI prevalence appraisal tool was used to assess the methodological quality of included studies (Munn et al. 2019).

### 2.1 Literature Search

A search strategy was developed following initial consultation with the school librarian. First, search terms were selected to identify published papers reporting on MNC prevalence data in acute care settings. Next, full‐text searches were conducted using the following key search terms: (‘missed nursing care’ OR ‘rationing of nursing’ OR ‘task left undone’ OR ‘unfinished nursing care’ OR ‘omitted nursing care’ OR ‘care left undone’) AND (‘patient’/exp OR patient OR ‘nurse’/exp OR nurse). Five electronic databases were searched (Embase, CINAHL, PubMed, Medline and Scopus) with search strategies adapted where necessary for each database. This was followed by word analysis of titles, abstracts and keywords used in describing each paper. To obtain up‐to‐date published literature, the authors performed two searches in the selected databases using the same search strategy. The first search was performed to select studies prior to 20th November 2021, and the second search was conducted on 14th December 2022, to include studies published from January 2021 to December 2022. Records identified were extracted to Rayyan (Ouzzani et al. 2016) for screening and then uploaded in Endnote (Bramer et al. 2017).

### 2.2 Inclusion and Exclusion Criteria

This review included primary MNC studies that involved patients and nurses (new graduates, experienced nurses, nurse managers, licensed practical nurses, enrolled nurses and nurse assistants in acute care settings), reports of the types and extent of MNC in acute care settings using a validated MNC instrument (Palese et al. 2021). Additionally, the included studies were performed in English language. Studies performed in maternal and child health settings, neonatal intensive care and paediatric care units were excluded.

### 2.3 Study Selection

The first author performed initial assessment of titles and abstracts of documents retrieved based on the inclusion and exclusion criteria and obtained the required full texts for further assessment. Then, independently, two authors reviewed documents and made the final decisions. Rayyan software was used for the above processes. The reasons for exclusion were recorded, and conflicts between the two reviewers were discussed and resolved with a third reviewer.

### 2.4 Assessment of Methodological Quality

The quality of studies was assessed using the Joanna Briggs Institute Critical Appraisal Checklist for Studies Reporting Prevalence Data based on nine items measuring appropriateness of sample frame, study participants, sample size, study subjects, data analysis, valid methods, condition of measurement, appropriate statistical analysis and appropriate response rate (Munn et al. 2015). Each of the nine items was measured on a four‐point Likert scale (yes = 3 points, unclear = 2, no = 2 points or not applicable = 0). Total scores for each of the studies were further converted into percentages to indicate level of quality (strong = > 80%, good = 70%–80%, adequate = 50%–69% and limited = < 50%) (Byeon 2019). Data analysis was guided by the approah described by (Borges Migliavaca et al. 2020).

### 2.5 Data Extraction and Statistical Analysis

The categories of information extracted from the data included author and publication year, country, economy, objective, design, setting, number of participants, MNC tool and factors influencing MNC. Nurse‐reported types and extent of MNC data were exported to JBI SUMARI for meta‐analysis. The metadata were further grouped under the following 10 subgroups for analysis: ambulation, mouthcare, emotional support, bathing, feeding, medication, documentation, discharge planning, hand washing and vital signs. The variance of the prevalence measures was stabilised using the Freeman–Tukey type arcsine square root transformation. Estimates for MNC prevalence were weighted and pooled by the inverse variance using a random‐effects model. A random model was used to account for sampling variation. The heterogeneity between the studies was assessed by using the *I* ^2^ test statistic (percentage of total variability between studies due to heterogeneity) and *p* values (*p* < 0.05 was considered statistically significant). The heterogeneity was considered as high, moderate or low when *I* ^2^ test statistic results were 75%, 50% and 25%, respectively. Expected heterogeneity in effect size across studies was related to differences in tools, populations and settings.

## 3 Results

### 3.1 Search Results

**FIGURE 1:**
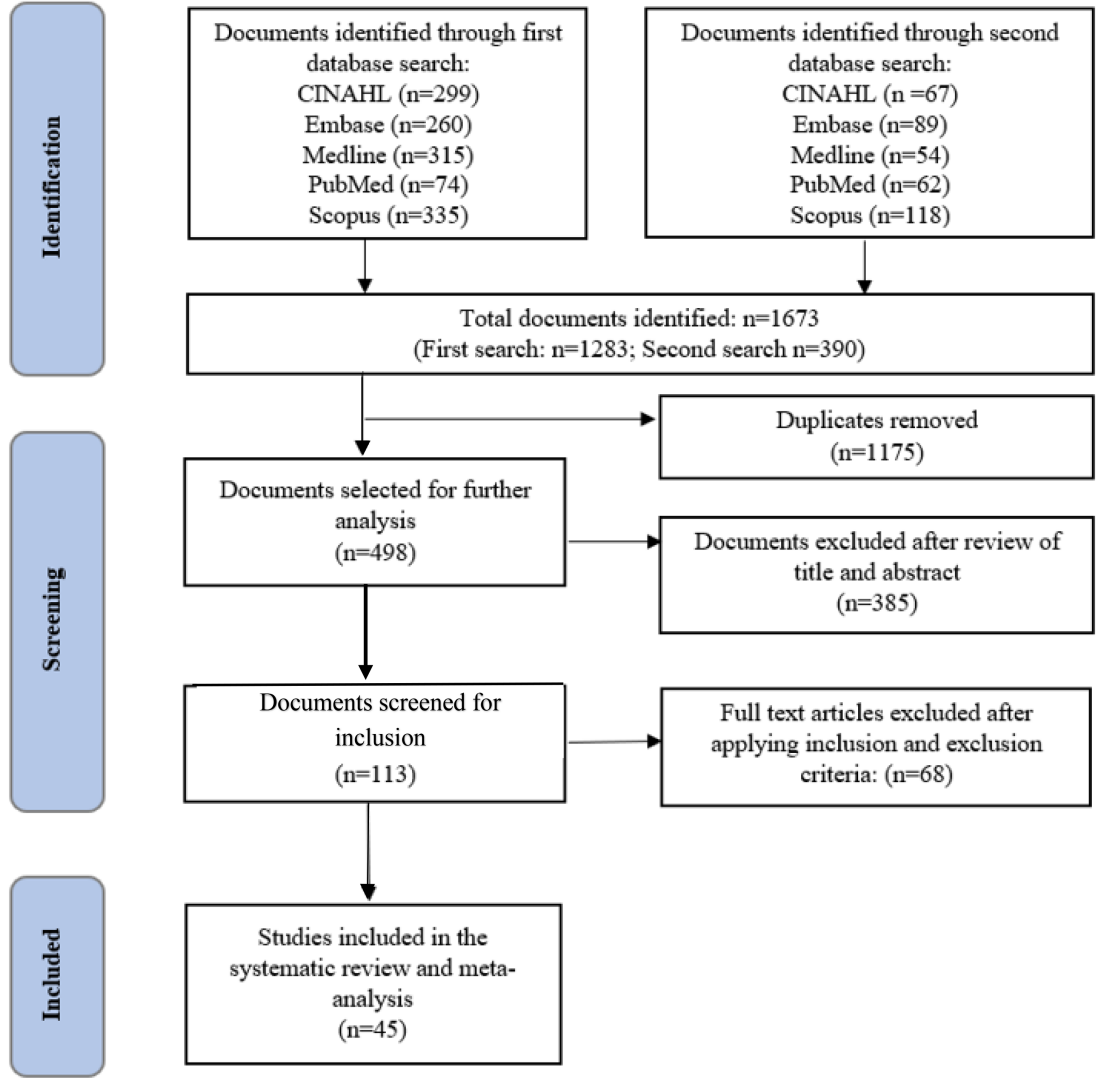
PRISMA flow diagram of selection of studies.

The PRISMA flow chart describes the literature search and articles selection process for this study (Figure 1). A total of 1673 documents were retrieved from five databases. After removing duplicates and unrelated studies, a total of 113 articles remained for full‐text review. The careful full‐text review of these studies led to the removal of 68 studies; thus, a total of 45 observational studies were finally included in this review.

### 3.2 Results of Quality Assessment

The quality of eligible studies was determined based on the JBI critical appraisal checklist, as presented in Table 1. According to the results, all studies had good or high‐quality scores, either adequate, good or strong. The most missed classification item was appropriate management of low response rate. All reviewed studies were included regardless of their quality.

**TABLE 1:** Critical appraisal results of the eligible studies.

No	Study	Q1	Q2	Q3	Q4	Q5	Q6	Q7	Q8	Q9	Score (%)
1	Al Muharraq et al. 2022	Y	Y	Y	Y	Y	Y	Y	Y	N	92.6
2	Al‐Kandari and Thomas 2009	Y	Y	N	N	N	Y	Y	Y	Y	77.7
3	Albsoul et al. 2022	Y	Y	N	Y	N	Y	Y	Y	N	77.7
4	Ausserhofer et al. 2014	Y	Y	Y	Y	Y	Y	Y	Y	Y	100
5	Ball et al. 2016	Y	Y	Y	Y	Y	Y	Y	Y	Y	100
6	Ball et al. 2014	Y	Y	Y	Y	Y	Y	Y	Y	Y	100
7	Campbell et al. 2020	Y	Y	Y	Y	Y	Y	Y	Y	N	92.6
8	Chapman et al. 2016	Y	Y	Y	Y	Y	Y	Y	Y	Y	100
9	Chegini et al. 2020	Y	Y	N	Y	Y	Y	Y	Y	Y	92.6
10	Cho et al. 2016	Y	Y	Y	Y	Y	Y	Y	Y	Y	100
11	Cho et al. 2019	Y	Y	Y	Y	Y	Y	Y	Y	Y	100
12	Du et al. 2020	Y	Y	Y	Y	Y	Y	Y	Y	Y	100
13	Duffy et al. 2018	Y	N	Y	Y	Y	Y	Y	Y	Y	92.6
14	Eskin Bacaksiz et al. 2020	Y	N	Y	Y	Y	Y	Y	Y	N	85.2
15	Friese et al. 2013	Y	Y	Y	Y	Y	Y	Y	Y	Y	100
16	Gravlin et al. 2010	Y	Y	Y	Y	Y	Y	Y	Y	N	92.6
17	Gurková et al. 2022	Y	Y	Y	Y	Y	Y	Y	Y	Y	100
18	Hernández‐Cruz et al. 2017	Y	Y	Y	N	Y	Y	Y	Y	N	85.2
19	Hosseini et al. 2022	Y	Y	N	Y	Y	Y	Y	Y	Y	92.6
20	Jarošová and Zeleníková 2019	Y	N	N	N	Y	Y	Y	Y	Y	77.7
21	Jones 2015	Y	Y	Y	Y	Y	Y	Y	Y	N	92.6
22	Kalánková et al. 2022	Y	Y	Y	Y	Y	Y	Y	Y	Y	100
23	Kalisch et al. 2009	Y	Y	Y	Y	Y	Y	Y	Y	N	92.6
24	Kalisch et al. 2012	Y	Y	Y	Y	Y	Y	Y	Y	Y	100
25	Kalisch et al. 2011	Y	Y	Y	Y	Y	Y	Y	Y	Y	100
26	Kalisch et al. 2014	Y	Y	Y	Y	Y	Y	Y	Y	Y	100
27	Kołtuniuk et al. 2021	Y	Y	Y	N	Y	Y	Y	Y	Y	92.6
28	Lucero et al. 2009	Y	Y	Y	Y	Y	Y	Y	Y	Y	100
29	Maloney et al. 2015	Y	N	Y	Y	Y	Y	Y	Y	N	85.2
30	Moreno‐Monsiváis et al. 2015	Y	Y	Y	Y	Y	Y	Y	Y	Y	100
31	Nymark et al. 2022	Y	Y	Y	Y	Y	Y	Y	Y	Y	100
32	Obregón‐Gutiérrez et al. 2022	Y	Y	N	N	Y	Y	Y	Y	N	85.2
33.	Orique et al. 2016	Y	N	N	Y	Y	Y	Y	Y	N	77.7
34	Palese et al. 2015	Y	Y	Y	Y	Y	Y	Y	Y	Y	100
35	Papastavrou et al. 2014	Y	Y	Y	Y	Y	Y	Y	Y	Y	100
36	Peterson et al. 2021	Y	Y	N	Y	Y	Y	Y	Y	N	85.2
37	Plevová et al. 2021	Y	Y	N	Y	Y	Y	Y	Y	N	85.2
38	Schubert et al. 2013	Y	Y	Y	Y	Y	Y	Y	Y	Y	100
39	Smith et al. 2018	Y	Y	Y	Y	Y	Y	Y	Y	Y	100
40.	Srulovici and Drach‐Zahavy 2017	Y	Y	Y	Y	Y	Y	Y	Y	Y	100
41	Taskiran Eskici and Baykal 2022	Y	Y	Y	Y	Y	Y	Y	Y	Y	100
42	Uchmanowicz et al. 2020	Y	Y	Y	Y	Y	Y	Y	Y	Y	100
43	Villamin et al. 2019	Y	Y	Y	Y	Y	Y	Y	Y	Y	100
44	von Vogelsang et al. 2021	Y	Y	N	Y	Y	Y	Y	Y	N	85.2
45	Winsett et al. 2016	Y	Y	N	Y	Y	Y	Y	Y	N	85.2

*Note:* JBI critical appraisal checklist for prevalence (the following 9 items):

Q1 = Was the sample frame appropriate to address the target population?

Q2 = Were study participants recruited in an appropriate way?

Q3 = Was the sample size adequate?

Q4 = Were the study subjects and setting described in detail?

Q5 = Was data analysis conducted with sufficient coverage of the identified sample?

Q6 = Were valid methods used for the identification of the condition?

Q7 = Was the condition measured in a standard, reliable way for all participants?

Q8 = Was there appropriate statistical analysis?

Q9 = Was the response rate adequate, and if not, was the low response rate managed appropriately?

Abbreviations: N = no, NA = not applicable, U = unclear, Y = yes.

### 3.3 Characteristics of the Included Studies

The overall characteristics of studies included in the analysis are summarised in Table 2. The studies were conducted across the globe and published from 2009 to 2022. The majority of the studies were performed in North America (*n* = 17) and Europe (*n* = 16) and the remaining in Asia (*n* = 10) and Australian regions (*n* = 2). Thirty‐five studies were conducted in two or more hospitals, and the remaining were in single centres. The number of hospitals included in multicentre studies ranged from 2 to 488. The studies were conducted in health facilities such as teaching, urban, rural and community hospitals. Most of the studies were cross‐sectional, with one being a descriptive case study. The study results showed that different tools have been used to report MNC, and the most common tool was the MISSCARE survey, which has both nurse and patient tools but different items. In this study, only nurse‐reported MNC data are presented in the meta‐analysis. In detail, of the 45 studies, only three studies measured patient reports of MNC, and these studies used the patient version of the MISSCARE survey (Albsoul et al. 2022; Kalisch, Lee, and Dabney 2014; Kalisch, Xie, and Dabney 2014; Moreno‐Monsiváis et al. 2015) (two studies reported from both nurses and patients).

**TABLE 2:** Characteristics of the studies and participants included.

	Author(s) and year	Country	Objective	Design	Setting	Number of participants	MNC tool	Main outcomes/factors associated with MNC
1	Al Muharraq et al. 2022	Saudi Arabia	To explore most common types and reasons behind MNC	Cross‐sectional	10 hospitals	Nurses = 604	MISSCARE	Inadequate labour resource was associated MNC. Labour resources influenced nurse job satisfaction and intention to leave.
2	Al‐Kandari and Thomas 2009	Kuwait	To assess MNC elements, contributing factors and relationship with staffing	Exploratory study	5 hospitals	Nurses = 780	TU	Increased workload, nurse age, work experience and educational background influenced MNC
3	Albsoul et al. 2022	Australia	To investigate the nature of MNC and influencing factors	Descriptive case study	1 hospital	Patient = 30 Nurses = 28	MISSCARE	Inadequate number of staff was associated with MNC
4	Ausserhofer et al. 2014	Belgium, England, Finland, Germany, Greece, Ireland, the Netherlands, Norway, Poland, Spain, Sweden and Switzerland	To describe prevalence and patterns of MNC	Cross‐sectional design	488 hospitals in 12 European countries	Nurses = 33 659	TU	Nurse related organisational factors such as unfavourable hospital environment, higher patient‐to‐nurse ratios and professional experience were associated with MNC
5	Ball et al. 2016	Sweden	To determine factors associated with MNC	Cross‐sectional survey	79 hospitals	Nurses = 10 174	TU	Nurses' role characteristics such as workload, shift type and patient dependency levels were associated with MNC
6	Ball et al. 2014	England	To examine the nature and prevalence of MNC by nurses	Cross‐sectional survey	46 hospitals	Nurses = 2917	TU	Inadequate nurse staffing was associated with MNC
7	Campbell et al. 2020	USA	To discover the extent of and factors associated with MNC	Cross‐sectional study	Alabama Hospitals (number of hospitals not reported)	Nurses = 950	PIRNCA	Increased nurse workload was associated with MNC.
8	Chapman et al. 2016	Australia	To investigate effects of teamwork on MNC	Descriptive exploratory study	4 hospitals	Nurses = 334	MISSCARE	Teamwork influenced with MNC
9	Chegini et al. 2020	Iran	To determine MNC prevalence and reasons for its occurrence	Cross‐sectional	8 hospitals	Nurses = 215	MISSCARE	Human resources, material resource and communication influenced MNC
10	Cho et al. 2016	South Korea	To explore association of nurse staffing, overtime, quality of care and MNC	Cross‐sectional	51 hospitals	Nurses = 3037	BERNCA	Inadequate nurse staffing was associated with MNC
11	Cho et al. 2019	South Korea	To examine the relationship among staffing, prioritisation of nursing activities, MNC, quality care and nurse outcomes	Cross‐sectional design	49 hospitals	Nurses = 2114	MISSCARE	Inadequate nurse staffing influenced MNC
12	Du et al. 2020	China	To identify the risk of MNC and contributing factors	Cross‐sectional study	34 hospitals	Nurses = 6158	MISSCARE	Inadequate human resource issues and increased workload influenced MNC
13	Duffy et al. 2018	USA	To describe and evaluate the factors associated with MNC	Cross‐sectional study	1 hospital	Nurses = 138	MISSCARE	Inadequate staffing, reduced satisfaction with current position, and collegial nurse‐physician relationships were associated with MNC
14	Eskin Bacaksiz et al. 2020	Turkey	To analyse MNC in private hospitals	Cross‐sectional	25 hospitals	Nurses = 897	MISSCARE	Long working hours and inadequate number of nurses were associated with MNC
15	Friese et al. 2013	USA	To quantify the degree of MNC in oncology units and compare between oncology and nononcology units	Cross‐sectional study	9 hospitals	Nurses = 2318 (352 oncology and 1966 nononcology nurses)	MISSCARE	Inadequate staffing was associated with MNC
16	Gravlin et al. 2010	USA	To measure nurses and nurse assistants reports of MNC	Quantitative descriptive design	3 hospitals	Registered nurses = 241 Nurse assistants (NAs) = 99	MISSCARE	Unexpected rise in patient volume and inadequate staffing influenced MNC
17	Gurková et al. 2022	Czech Republic	To investigate which domains of work environment are significant predictors of MNC during COVID‐19	Cross‐sectional	4 hospitals	Nurses = 371	MISSCARE	Working overtime and work environment quality influenced MNC
18	Hernández‐Cruz et al. 2017	Mexico	To determine the factors that influence MNC	Cross‐sectional	1 hospital	Nurses = 71	MISSCARE	Human resource factors were associated with MNC
19	Hosseini et al. 2022	Iran	To investigate MNC and the reasons for its occurrence during coronavirus disease	Cross‐sectional	Educational hospitals (number of hospitals not reported)	Nurses = 135	MISSCARE	Urgent patient situations, inadequate staff and unexpected rise in patient volume and/or patient acuity on the unit were associated with MNC
20	Jarošová and Zeleníková 2019	Czech	To investigate the amount, type and reasons for MNC	Cross‐sectional study	2 hospitals	Nurses = 100	PIRNCA	Inadequate number of nursing staff was associated with MNC
21	Jones et al. 2015	USA	To examine the phenomenon of MNC	Cross‐sectional	11 Texas Health and Human Services Regions (number of hospitals not reported)	Nurses = 226	PIRNCA	Time scarcity was associated with increased risk of MNC
22	Kalánková et al. 2022	Slovak Republic	To examine the association between selected hospital, unit and staff variables and the prevalence of MNC	Cross‐sectional	7 hospitals	Nurses = 895	PIRNCA	Job satisfaction and work environment factors such as unit type, shift type and education experience influenced MNC
23	Kalisch et al. 2009	USA	To examine why and what nursing care is missed	Cross‐sectional study	3 hospitals	Nurses = 459	MISSCARE	Inadequate labour resources, inadequate material resources and communication factors influenced MNC
24	Kalisch et al. 2012	USA	To test the perceptions of nurse leaders and nurse staff members extent and type of MNC	Cross‐sectional study	11 hospitals	Nursing staff = 4415 Nurse leaders = 104	MISSCARE	Inadequate material and labour resources influenced MNC
25	Kalisch et al. 2011	USA	To report on the extent and type of MNC and the reasons for MNC	Cross‐sectional	10 hospitals	Nurses = 3143 Nurse Assistants = 943	MISSCARE	Labour resources, material resources and communication factors influenced MNC
26	Kalisch et al. 2014	USA	To determine the extent and type of MNC	Cross‐sectional study	2 hospitals	Patients = 729	MISSCARE	Patients who reported adverse events (such as skin breakdown/pressure ulcers, medication errors, new infections, intravenous fluid running dry) reported more MNC
27	Kołtuniuk et al. 2021	Poland	To assess MNC among nurses	Cross‐sectional	2 hospitals	Nurses = 529	BERNCA‐R	Patient‐to‐nurse ratio and level of job satisfaction influenced MNC
28	Lucero et al. 2009	USA	To describe nurses reports of MNC	Secondary analysis of survey data	168 hospitals	Nurses = 10 184	TU	Variations in care environment and process of care were associated with MNC
29	Maloney et al. 2015	USA	To measure frequency types and reasons for MNC	Descriptive study	3 hospitals	Nurses = 205	MISSCARE	Inadequate labour and material resources influenced MNC
30	Moreno‐Monsiváis et al. 2015	Mexico	To determine MNC in patients and factors related to missed care, based on nurses‐patient perception	Descriptive correlational study	1 hospital	Nurses= 160 Patients= 160	MISSCARE	Human resources, material resources and communication factors influenced MNC
31	Nymark et al. 2022	Sweden	To evaluate missed MNC and patient safety during the first wave of the COVID‐ 19 pandemic	Cross‐sectional	1 hospital	‐Nurses = 43 ‐NAs = 59	MISSCARE	Increased reported MNC in activities such as wound care and basic nursing care among COVID‐19 sample compared to non‐COVID group.
32	Obregón‐Gutiérrez et al. 2022	Spain	To analyse the quality of care provided during the COVID‐19 pandemic.	Retrospective cross‐sectional	1 hospital	Nurses = 225	BERNCA	Personal and professional characteristics influenced MNC
33.	Orique et al. 2016	USA	To identify aspects of MNC and their relationship to unit workload.	Cross‐sectional study	1 hospital	Nurses = 169	MISSCARE	Labour resource, materials resources and communication factors influenced MNC
34	Palese et al. 2015	Italy	To identify amount, types and reasons for MNC	Cross‐sectional	12 hospitals	Nurses = 314	MISSCARE	Length of experience in unit, communication, full time job and timeliness to care influenced MNC
35	Papastavrou et al. 2014	Cyprus	To examine MNC and the relationship between work environment and MNC	Cross‐sectional	All public hospitals (number not reported)	Nurses = 393	BERNCA	Teamwork, leadership and autonomy and staff communication influenced MNC
36	Peterson et al. 2021	Estonia	To describe care MNC and relationship to organisational characteristics	Cross‐sectional	5 hospitals	Nurses = 169	BERNCA	Nurses' role influenced MNC as participants did not consider activities of daily living to be task required to be completed by nurses
37	Plevová et al. 2021	Czech Republic	To explore the frequency of MNC and relationship with nurse's job satisfaction	Cross‐sectional	9 hospitals	Nurses = 513	MISSCARE	Job satisfaction was associated with MNC
38	Schubert et al. 2013	Switzerland	To describe MNC levels and potential predictors	Cross‐sectional	35 hospitals	Nurses = 1633	BERNCA	Inadequate staffing and safety climate influenced MNC
39	Smith et al. 2018	USA	To understand how MNC is associated with nurse work environment	Cross‐sectional study	5 hospitals	Nurses = 233	MISSCARE	Nurse work environments and collective efficacy influenced MNC
40.	Srulovici and Drach‐Zahavy 2017	Israel	To test the joint effects of personal and ward accountability on MNC	Cross‐sectional	8 hospitals	Nurses = 295	MISSCARE	Nurse ward accountability influenced MNC
41	Taskiran Eskici and Baykal 2022	Turkey	To examine MNC frequency, reasons, correlates and predictors	Cross‐sectional	10 hospitals	Nurses = 1310	MISSCARE	Work environment factors such as nurse–patient ratio and teamwork influenced MNC
42	Uchmanowicz et al. 2020	Poland	To assess the impacts of burnout and job satisfaction on MNC	Cross‐sectional	1 hospital	Nurses = 594	BERNCA	Burnout and job satisfaction influenced MNC
43	Villamin et al. 2019	USA	To evaluate the difference in perceived MNC occurrence	Descriptive study	1 hospital	Nurses = 286 (baseline)	MISSCARE	The implementation of primary team nursing did not influence MNC, however unit type influenced MNC
44	von Vogelsang et al. 2021	Sweden	To evaluate frequency, types and reasons for MNC during the COVID‐ 19 pandemic	Comparative Cross‐sectional	1 hospital	Nurses COVID sample = 130 Reference sample = 157	MISSCARE	Inadequate staffing and skill mix, influenced MNC
45	Winsett et al. 2016	USA	To explore work environment by evaluating nurse reported MNC	Descriptive correlational	4 hospitals	Nurses = 168	MISSCARE	Unexpected rise in patient volume, heavy admissions/discharges, inadequate staffing and inadequate material resources influenced MNC

Abbreviations: BERNCA = Basel Extent of Rationing of Nursing Care, MISSCARE, Missed Nursing Care Survey, MNC = missed nursing care, NA = nurse assistant, PIRNCA = Perceived Implicit Rationing of Nursing Care.

### 3.4 Missed Care Prevalence, Types and Associated Factors

To estimate the pooled prevalence of the types of MNC, the results of the meta‐analysis were obtained using a random effect model of 44 studies with nurse participants. The estimated prevalence of the top 10 MNC activities is reported in Table 3. Figures 2, 3, 4, 5, 6, 7, 8, 9, 10, 11 show the forest plot of MNC activities. Overall, heterogeneity among studies was high.

**TABLE 3:** Prevalence rates, confidence intervals and heterogeneity indices for top 10 activities missed.

Activity	Prevalence (%)	95% CI	Heterogeneity (*I* ^2^)
Ambulation	45.8	(0.37, 0.55)	99.6
Mouth care	35.9	(0.30, 0.43)	99.7
Emotional support	33.2	(0.26, 0.41)	99.5
Bathing	30.8	(0.22, 0.41)	99.6
Feeding	30.0	(0.23, 0.38)	99.5
Medication	28.5	(0.22, 0.36)	99.7
Documentation	26.9	(0.27, 0.27)	99.7
Hand washing	18.2	(0.12, 0.25)	99.3
Discharge planning	17.8	(0.18, 0.18)	99.3
Vital signs	10.5	(0.10, 0.11)	99.2

**FIGURE 2:**
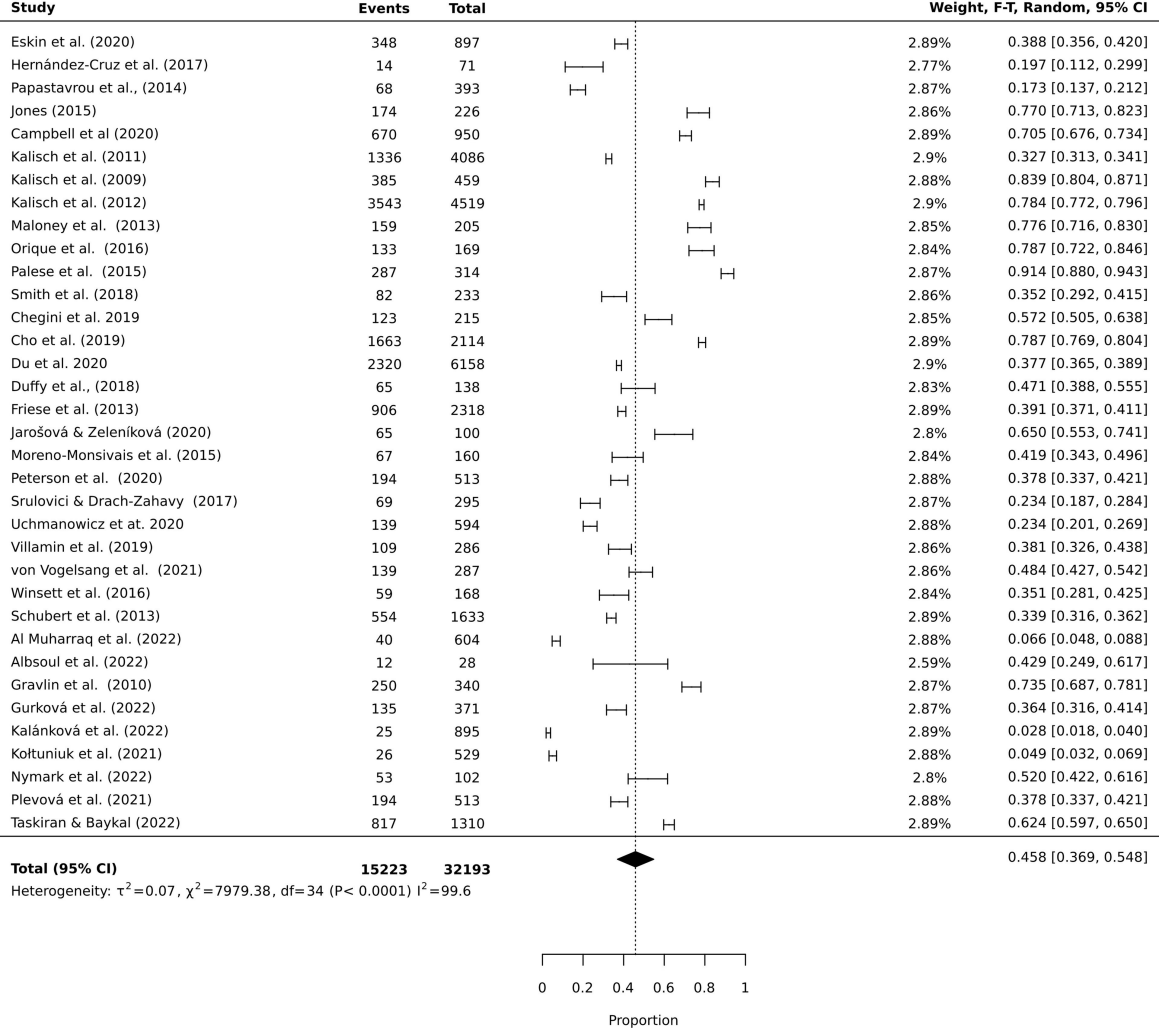
Forest plot estimating global prevalence of nurse‐reported missed ambulation.

**FIGURE 3:**
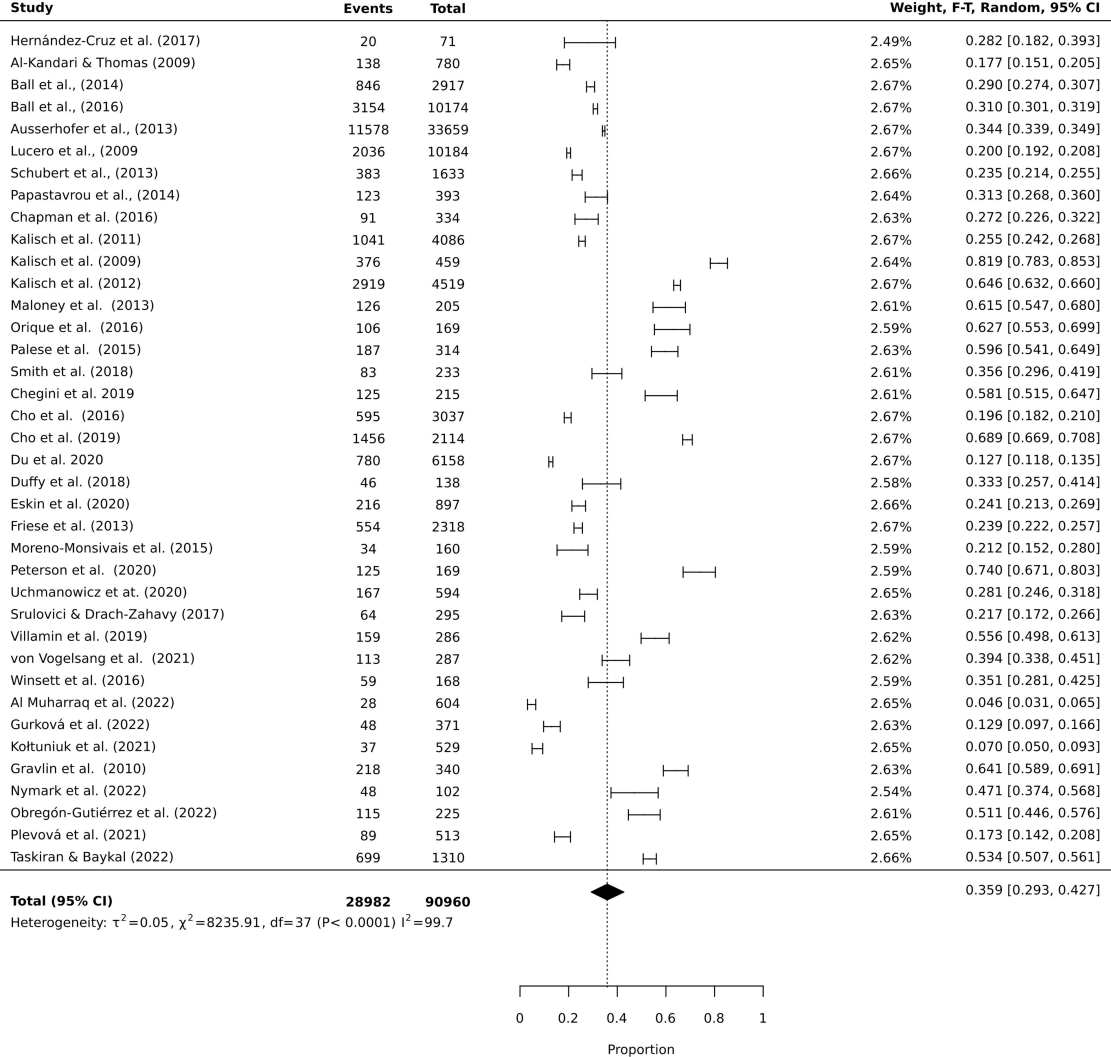
Forest plot estimating global prevalence of nurse‐reported missed mouthcare.

**FIGURE 4:**
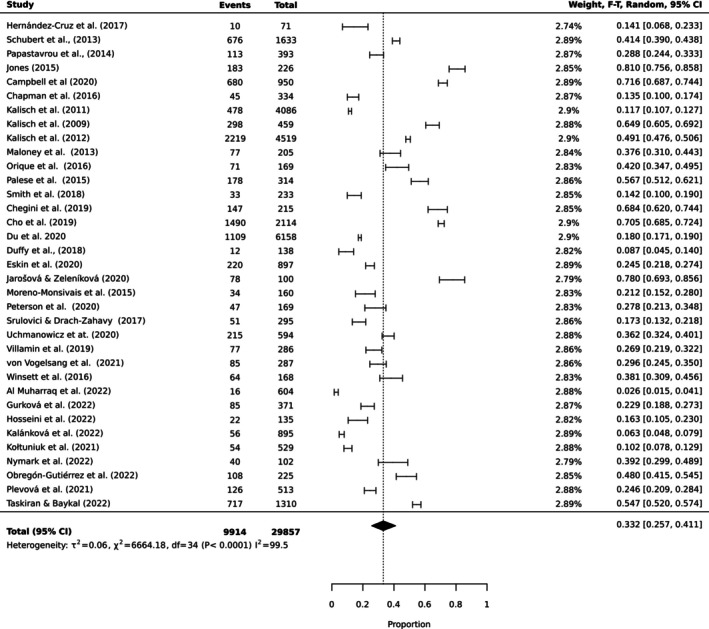
Forest plot estimating global prevalence of nurse‐reported missed emotional support.

**FIGURE 5:**
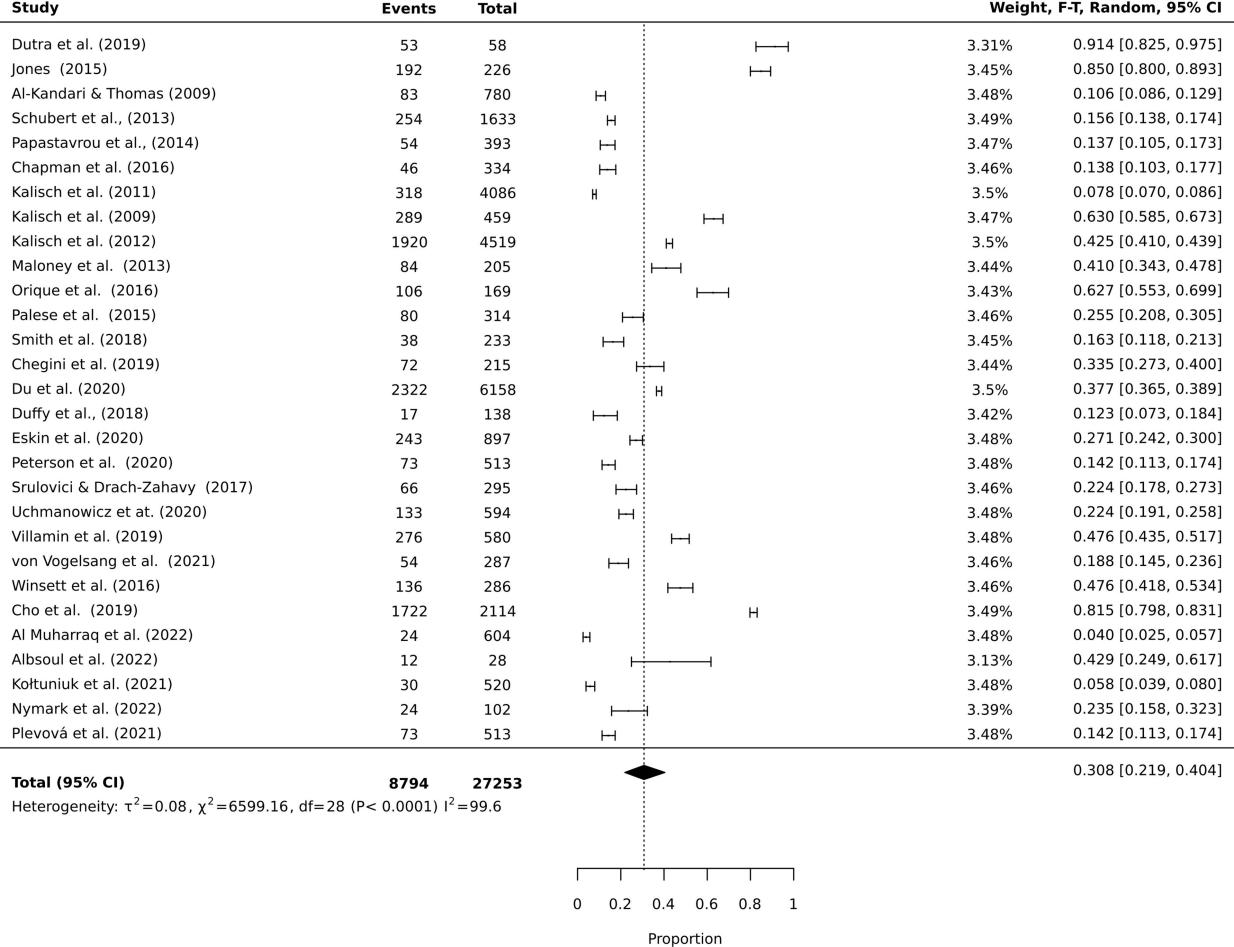
Forest plot estimating global prevalence of nurse‐reported missed bathing.

**FIGURE 6:**
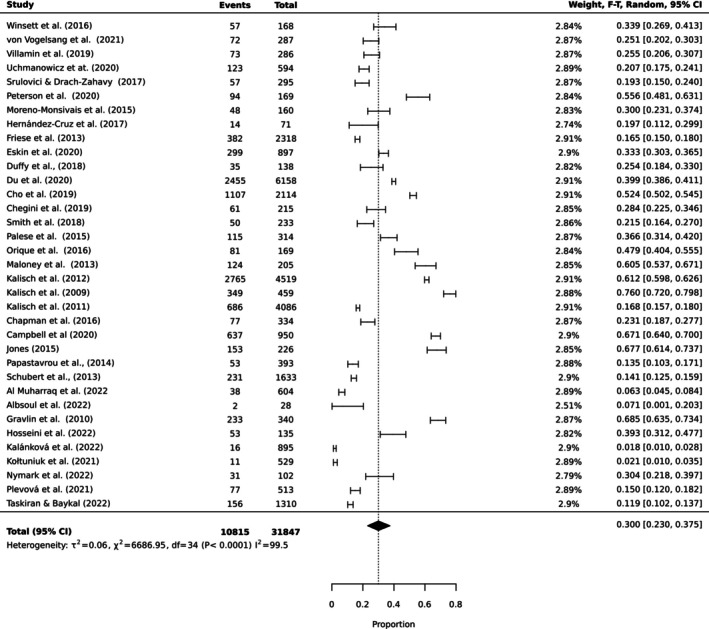
Forest plot estimating global prevalence of nurse‐reported missed feeding.

**FIGURE 7:**
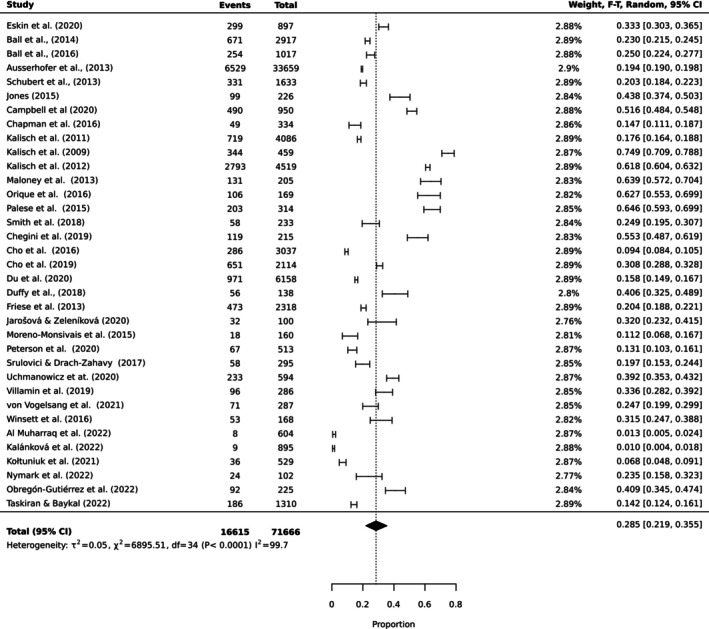
Forest plot estimating global prevalence of nurse‐reported missed medication.

**FIGURE 8:**
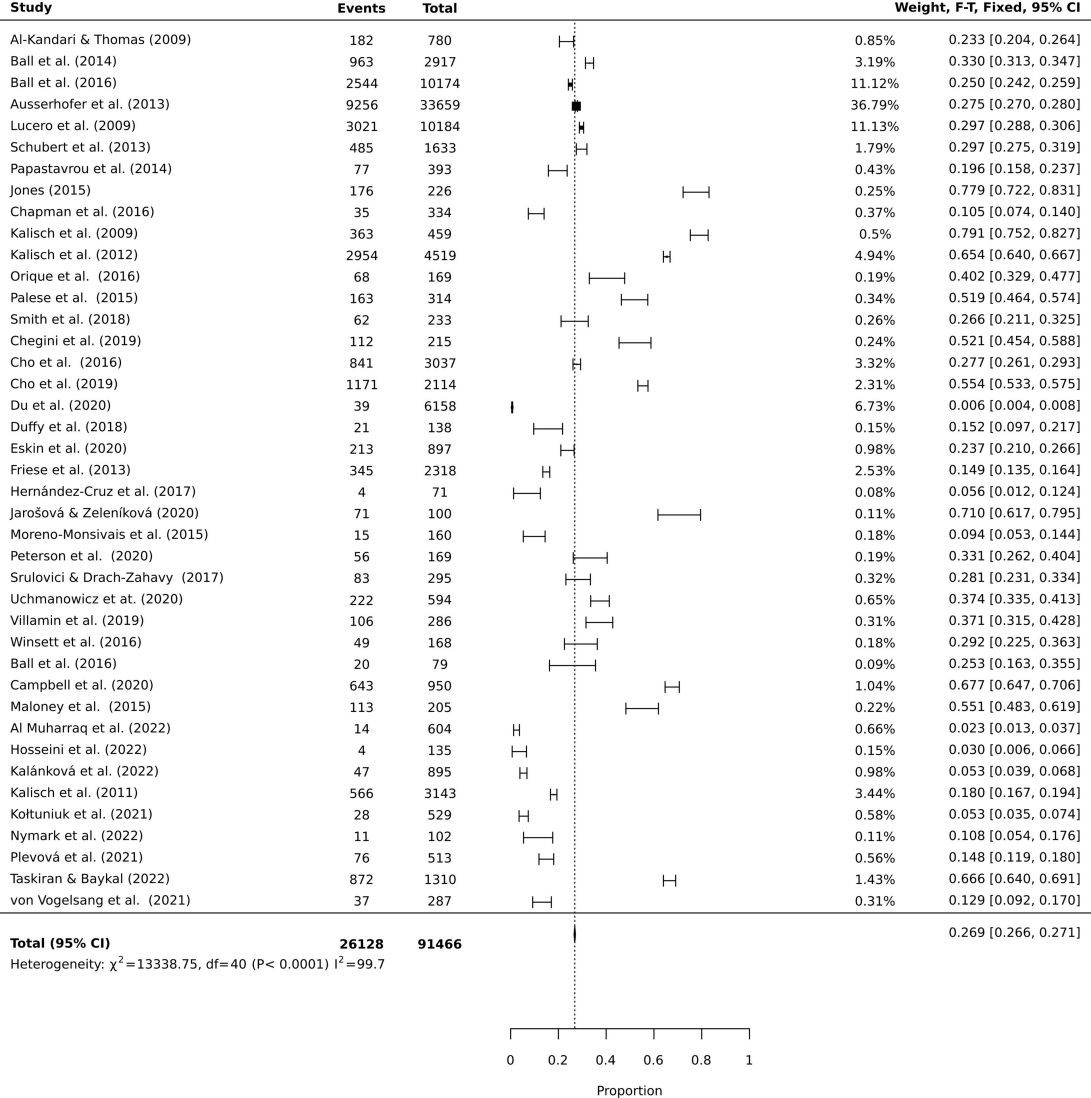
Forest plot estimating global prevalence of nurse‐reported missed documentation.

**FIGURE 9:**
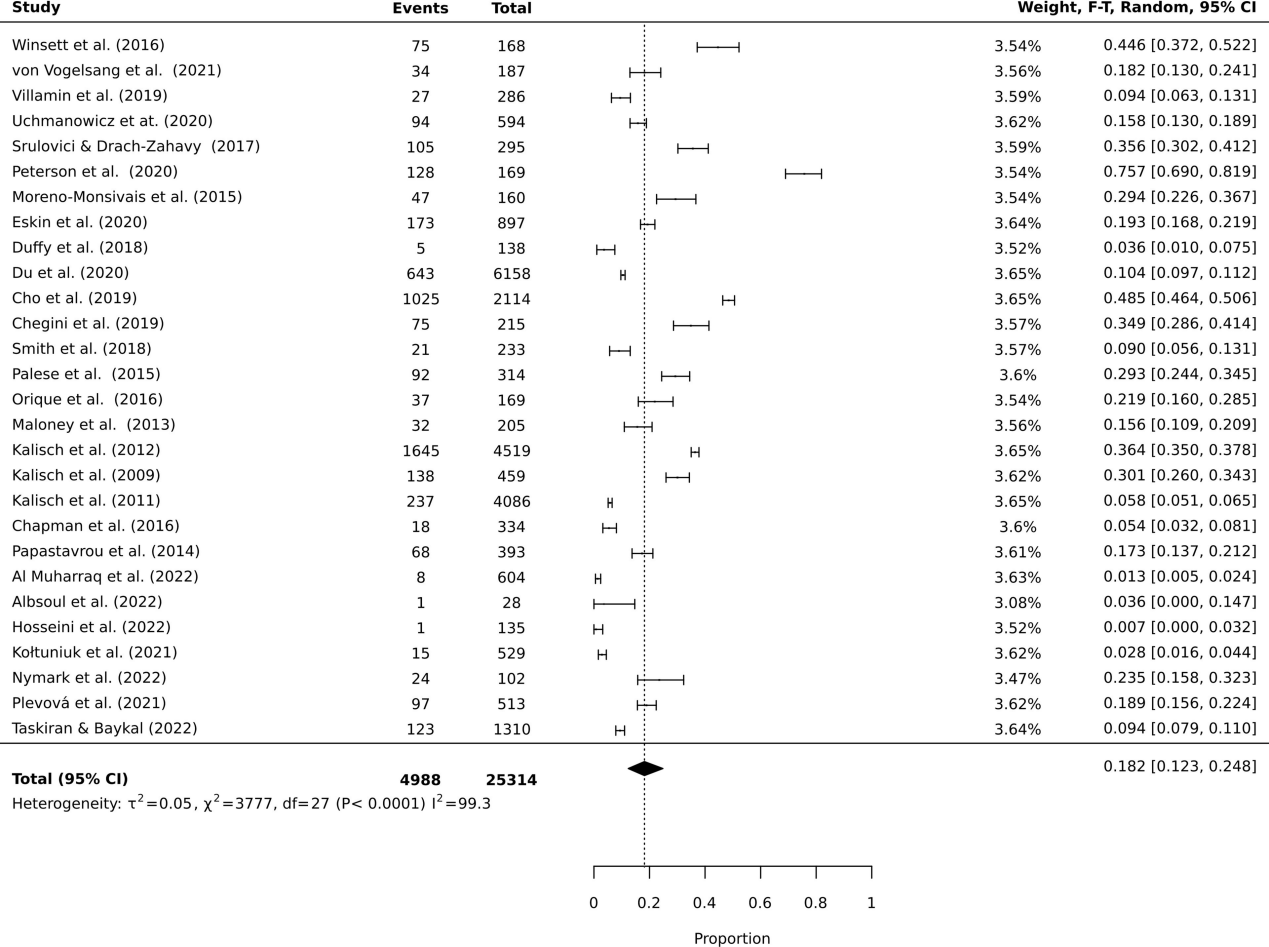
Forest plot estimating global prevalence of nurse‐reported missed hand washing.

**FIGURE 10:**
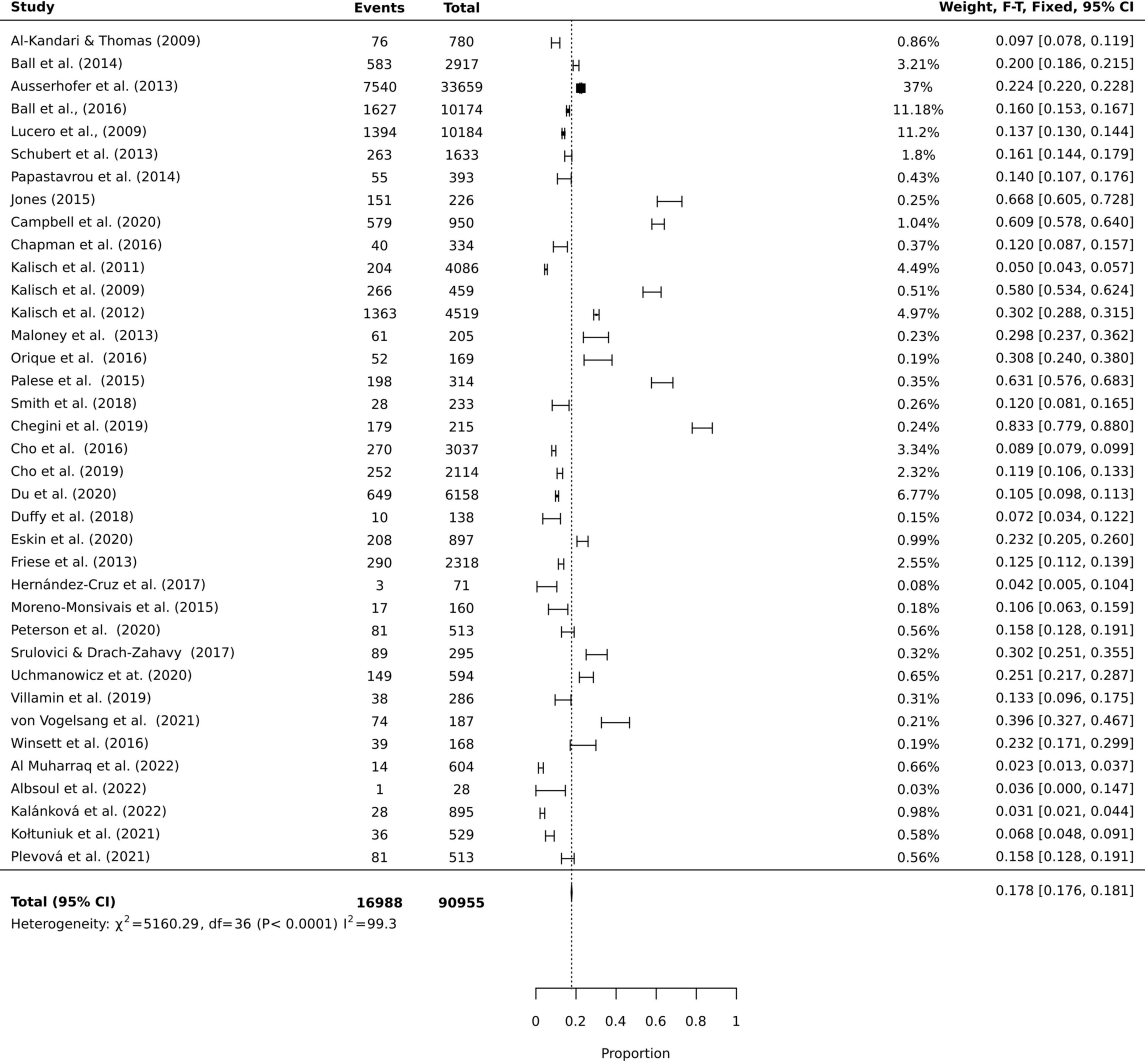
Forest plot estimating global prevalence of nurse‐reported missed discharge planning.

**FIGURE 11:**
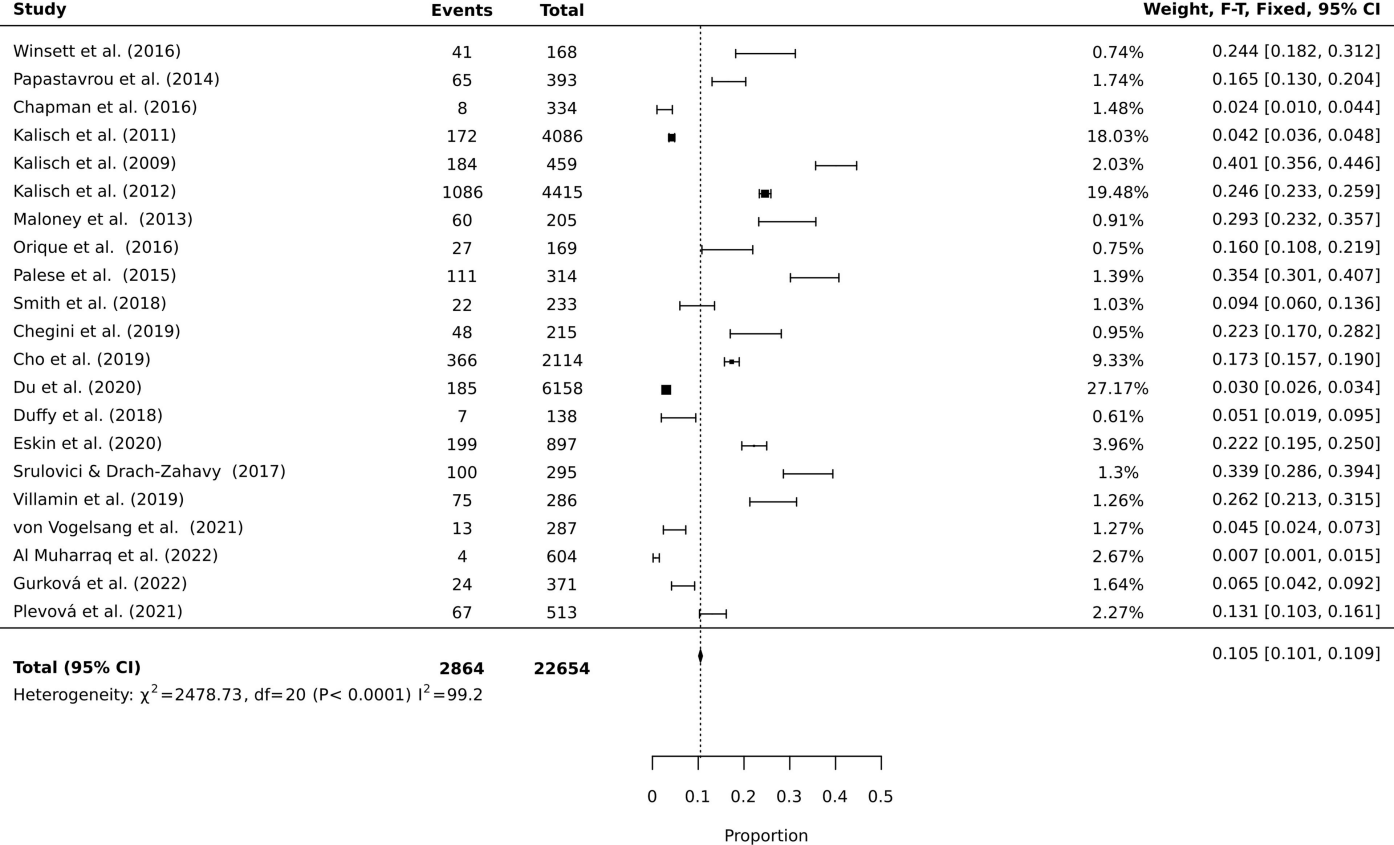
Forest plot estimating global prevalence of nurse‐reported missed vital signs.

Factors identified as associated with MNC mainly included reduced nurse workforce or labour resources, job satisfaction, intention to leave (Al Muharraq et al. 2022; Ball et al. 2016), nurse age, experience and educational background (Al‐Kandari and Thomas 2009), organisational factors (unfavourable hospital environment), material resources, patient dependency level, teamwork and communication (Ball et al. 2016; Campbell et al. 2020). Overall, among the 45 studies, inadequate staffing or limited labour resource was the most identified factor associated with MNC. In one study, nurse participants reported that activities of daily living were not considered as nursing tasks thus increasing the reported levels of MNC (Peterson et al. 2021).

## 4 Discussion

MNC remains a challenge for nurses, patients and the entire health system worldwide, even after 20 years of research in the area (Jones et al. 2021). This systematic review and meta‐analysis quantified the results of original research measuring the prevalence and associated factors of nurse–patient‐reported MNC. To the best of the researchers' knowledge, this is the first study attempting to estimate the prevalence of reported MNC across the globe using a meta‐analysis approach. The meta‐analysis performed in this study included 44 published studies, yielding a total of 141 312 nurse‐reported MNC events. Findings from this study support the evidence that significant nursing care activities are not being completed in acute care settings, and these fundamental nursing activities can lead to unsafe patient outcomes.

This finding is unsurprising given the widespread nurse staffing shortage (Peters 2023). A survey analysis among inpatients in 20 units of two acute care hospitals in the United States found that nurses frequently missed care such as mouthcare, ambulation, information about procedures and bathing (Kalisch, Lee, and Dabney, 2014) and that inability to complete patient care was associated with morbidity and mortality (Ball et al. 2018; Jones et al. 2015). In a literature review by Recio‐Saucedo and colleagues involving registered nurses and nurse assistants, most of the participants reported the existence of the MNC phenomenon and the potential impact of MNC on patient outcomes (Recio‐Saucedo et al. 2018). The study highlighted staff awareness of reported MNC and potentially dangerous consequences for hospitalised patients' missed care. Their review and other studies have further identified that understaffing, skill mix and unsafe patient outcomes contribute to reported MNC (Cho et al. 2020; Griffiths et al. 2018). Worth noting is the fact that MNC may not generate serious negative outcomes for younger active patients but can have dangerous consequences for older patients and produce a cascade of iatrogenesis (Bail and Grealish 2016; Thornlow et al. 2009).

Regarding the most frequently missed nursing activities, findings from this study showed that the top 10 most missed nursing activities included ambulation, mouthcare, emotional support, bathing and feeding. Less frequently missed activities were vital signs, discharge planning, hand hygiene, documentation and medication. Studies that have examined specific types of nursing activities corroborate these findings. For example, Kalisch, Xie, and Dabney (2014) in a literature review on the impact of inpatient mobilisation found ambulating a patient to be an important activity associated with safe hospitalisation outcomes. Regrettably, in a recent systematic review and meta‐analysis by Fazio et al. (2020) in acute care settings, their findings suggested that most hospitalised adult patients were primarily inactive and remained in bed throughout hospitalisation. While bedrest during hospitalisation can promote a reduction in oxygen consumption and metabolism, lack of ambulation has been associated with the rise in mortality, functional decline and cognitive impairment (Calero‐García et al. 2017). In terms of preventative measures (such as prevention of missed ambulation), research is still ongoing to monitor ambulation critically and determine a way to reduce missed ambulation, which has been reported globally because of insufficient nurse workforce (Longhini et al. 2021; Schubert et al. 2021). The significant finding of missed ambulation prevalence rate in this study is similar to previous research findings performed in this domain. This finding suggests that patients in acute care settings are in a potentially dangerous and volatile condition during hospitalisation, and this threatening situation is further compounded by a limited nurse workforce (Palese et al. 2015).

The second most frequently MNC activity found in acute care settings according to this meta‐analysis was mouthcare. Munro and Baker (2018) conducted a single arm intervention study in the United States using pre and post data to determine the effectiveness of mouthcare in the prevention of hospital acquired pneumonia. The authors found that although mouthcare seems to be a simple basic nursing care procedure, its omission impacts considerably on healthcare cost, quality of care and patients' overall wellbeing because oral care significantly reduces the risk of hospital‐acquired pneumonia onset. Furthermore, based on the positive outcomes of their study, eight centres in Virginia, United States, adopted the measurement of nonventilator hospital‐acquired pneumonia to gather evidence‐based data and develop interventions to maintain patient safety following hospitalisation. Additionally, their study inferred that the prevalence of undocumented daily missed oral care is a missed opportunity and adapting interventions to provide oral care can be an effective way to reduce hospital acquired pneumonia and save patients' lives. For patients 64 years and older who represent the largest hospitalised patient population, addressing their more complex care needs to reduce risk of cascaded iatrogenesis such as pressure sores, pneumonia and delirium is vital (Bail and Grealish 2016; Foley and Luz 2021).

Emotional support through nurse–patient communication emerged as the third most frequent missed activity. In a systematic review by He and colleagues, the authors demonstrated the importance of communication in reducing reported MNC (He et al. 2022). The authors highlighted the need for nurses to consider interventions to improve communication among healthcare teams and patients. In a qualitative study involving 20 nurses, the need for effective communication between nurses and hospitalised patients has been emphasized because good communication is critical for safe patient outcomes (Dithole et al. 2017).

It is worth noting that most of the top 10 reported MNC activities in this study were fundamental or basic care activities such as ambulation, mouthcare, emotional support, bathing and feeding. As nursing remains a holistic discipline that cares for the whole patient and not a part of a patient’s being, it remains critical for nurses, nurse managers, nurse academics and nurses in the policy workforce to relook at the profession and ask the questions ‘to what extent are nurses providing holistic nursing care amid the global phenomenon of missed care, unfinished nursing care or care left undone?’ Further cross‐disciplinary research is needed to uncover why each nursing activity is missed and the reasons for each missed care activity. It is possible that the main reasons for missed ambulation care in a ‘Magnet’ facility may be different from that in a private or rural hospital. Hence, interventions in reducing MNC may vary due to variations in facility types.

Longhini et al. (2021) proposed strategies to reduce frequency of MNC to advance the quality of patient care. The authors suggested the need for health workers and their patients to be more aware of the existing problem of errors of omission (care that is missed) and as a consequence, plan to develop and initiate quality improvement measures targeting specific missed activities. Schubert et al. (2021) have also argued that specific objective interventions such as fall prevention (through falls risk assessment), can have positive impact on selected activities and may offer an option for reducing MNC. However, the authors have cautioned that there is limited global evidence regarding targeted intervention to reduce specific types of nursing activities missed. Additionally, in another study by Griffiths et al. (2018), the authors recommended objective and digitalised measurement strategies to evaluate the impact of missed activities during hospitalisation. However, some authors have highlighted the fact that preventative approaches may not be ‘one size fit’, thus interventions can vary based on hospital unit characteristics (Hessels et al. 2019; Vryonides et al. 2018).

### 4.1 Limitations

One important limitation of this study is the self‐reporting method of MNC data collection from nurses, which may have introduced underreporting or overreporting of MNC events. Another limitation of this systematic review and meta‐analysis was that because patient reported MNC was different compared to nurse reported MNC, patient MNC events were not included in the meta‐analysis. Additionally, overall patient‐reported MNC was scanty. Future studies should be conducted investigating patient‐reported MNC. Furthermore, because MNC data were drawn mainly from cross‐sectional studies, a cause‐and‐effect relationship (between MNC and associated factors) cannot be inferred. Further research using different data collection methods such as direct nurse–patient care observation should be conducted.

## 5 Conclusion

This review provides a baseline assessment of the types of MNC activities, which can be used to compare future research and inpatient MNC practices. While there is substantial heterogeneity in how researchers conceptualize, define and measure MNC, there is consistent evidence that significant nursing activities are being missed during patient hospitalisation. In order to reduce and prevent MNC, we must first be able to monitor missed nursing activities such as ambulation, in a way that is accurate, clinically significant and does not increase workload to already heavy clinical workloads and required documentation burden.

Future research should establish standard methods for determining MNC activities and outcomes. Strengthening nursing care capacity, promoting active nurse participation in addressing MNC and establishing systems to prevent missed care can effectively reduce care gaps and associated unsafe outcomes (Longhini et al. 2021). The need for a more complete view of missed activities over time is important. Such efforts will advance the science of MNC and improve care based on data‐driven strategies.

## Author Contributions

All authors listed in this manuscript meet the authorship criteria and are in agreement with the content of this manuscript.

## Funding

This research was jointly supported by the School of Nursing and Midwifery, Edith Cowan University, and the Centre for Nursing Research, Sir Charles Gairdner Hospital, Perth, Australia. Afia Achiaa Sarpong is supported by the Sir Charles Gairdner Hospital Industry PhD Scholarship and the Edith Cowan University Higher Degree by Research fund.

## Conflicts of Interest

The authors declare no conflicts of interest.

## Data Availability

Data are available on request from the corresponding author.
